# Radiomics: a new tool to differentiate adrenocortical adenoma from carcinoma

**DOI:** 10.1093/bjsopen/zraa061

**Published:** 2021-03-03

**Authors:** F Torresan, F Crimì, F Ceccato, F Zavan, M Barbot, C Lacognata, R Motta, C Armellin, C Scaroni, E Quaia, C Campi, M Iacobone

**Affiliations:** Endocrine Surgery Unit, Department of Surgery, Oncology and Gastroenterology DISCOG, University Hospital of Padova, Padua, Italy; Radiology Unit, Department of Medicine DIMED, University Hospital of Padua, Padua, Italy; Endocrinology Unit, Department of Medicine DIMED, University Hospital of Padua, Padua, Italy; Radiology Unit, Department of Medicine DIMED, University Hospital of Padua, Padua, Italy; Endocrinology Unit, Department of Medicine DIMED, University Hospital of Padua, Padua, Italy; Radiology Department, University Hospital of Padua, Padua, Italy; Radiology Unit, Department of Medicine DIMED, University Hospital of Padua, Padua, Italy; Endocrine Surgery Unit, Department of Surgery, Oncology and Gastroenterology DISCOG, University Hospital of Padova, Padua, Italy; Endocrinology Unit, Department of Medicine DIMED, University Hospital of Padua, Padua, Italy; Radiology Unit, Department of Medicine DIMED, University Hospital of Padua, Padua, Italy; Department of Mathematics ‘Tullio Levi-Civita’, University of Padua, Padua, Italy; Endocrine Surgery Unit, Department of Surgery, Oncology and Gastroenterology DISCOG, University Hospital of Padova, Padua, Italy

## Abstract

**Background:**

The main challenge in the management of indeterminate incidentally discovered adrenal tumours is to differentiate benign from malignant lesions. In the absence of clear signs of invasion or metastases, imaging techniques do not always precisely define the nature of the mass. The present pilot study aimed to determine whether radiomics may predict malignancy in adrenocortical tumours.

**Methods:**

CT images in unenhanced, arterial, and venous phases from 19 patients who had undergone resection of adrenocortical tumours and a cohort who had undergone surveillance for at least 5 years for incidentalomas were reviewed. A volume of interest was drawn for each lesion using dedicated software, and, for each phase, first-order (histogram) and second-order (grey-level colour matrix and run-length matrix) radiological features were extracted. Data were revised by an unsupervised machine learning approach using the K-means clustering technique.

**Results:**

Of operated patients, nine had non-functional adenoma and 10 carcinoma. There were 11 patients in the surveillance group. Two first-order features in unenhanced CT and one in arterial CT, and 14 second-order parameters in unenhanced and venous CT and 10 second-order features in arterial CT, were able to differentiate adrenocortical carcinoma from adenoma (*P* < 0.050). After excluding two malignant outliers, the unsupervised machine learning approach correctly predicted malignancy in seven of eight adrenocortical carcinomas in all phases.

**Conclusion:**

Radiomics with CT texture analysis was able to discriminate malignant from benign adrenocortical tumours, even by an unsupervised machine learning approach, in nearly all patients.

## Teaser

This pilot study aimed to determine whether radiomics can predict malignancy in adrenocortical tumours. Two first-order features in unenhanced CT and one in arterial CT, and 14 second-order parameters in unenhanced and venous CT and 10 second-order features in arterial CT, were able to differentiate adrenocortical carcinoma from adenoma (*P* < 0.050). The unsupervised machine learning approach correctly predicted malignancy in seven of eight adrenocortical carcinomas in all phases. Radiomics with texture analysis can efficiently discriminate malignant from benign adrenocortical tumours in unenhanced and contrast-enhanced CT, even in an unsupervised machine learning approach.

## Resumen

**Antecedentes:**

El mayor reto en el tratamiento de los tumores adrenales “indeterminados” descubiertos incidentalmente es diferenciar entre lesiones benignas y malignas. En ausencia de claros signos de invasión o metástasis, las técnicas de imagen no siempre definen de forma precisa la naturaleza de la lesión. Este estudio piloto tuvo como objetivo determinar si ‘radiomics’ puede predecir la malignidad en los tumores adrenocorticales.

**Métodos:**

Se revisaron imágenes de tomografía computarizada (*computerized tomography*, CT) sin contraste, en fase arterial y en fase venosa de pacientes sometidos a resección de tumores adrenocorticales y de una cohorte que había recibido seguimiento durante al menos 5 años por ‘incidentalomas’, dibujando un volumen de interés para cada lesión con un software específico, para extraer de cada fase las características radiológicas de primer orden (histograma) y segundo orden (*grey level colour matrix and run length matrix*). Los datos fueron revisados mediante un aprendizaje automático no supervisado con la técnica de conglomerados de K medias.

**Resultados:**

De los pacientes operados, 9 tenían un adenoma no funcionante y 10 un carcinoma. Hubo 11 pacientes en el grupo de seguimiento. En total, 2 características de primer orden en el CT sin contraste y 1 en el CT en fase arterial; 14 parámetros de segundo orden en CT sin contraste y en fase venosa y 10 características de segundo orden en CT en fase arterial fueron capaces de diferenciar carcinoma adrenocortical de adenoma (*P* < 0,050). Después de excluir dos casos malignos atípicos (*outliers*), el abordaje con aprendizaje automático no supervisado predijo correctamente la malignidad en el 87,5% de carcinomas adrenocorticales en todas las fases.

**Conclusión:**

‘Radiomics’ con análisis de texturas del CT fue capaz de discriminar tumores adrenocorticales malignos de benignos, incluso en un abordaje con aprendizaje automático no supervisado, en casi todos los pacientes.

## Introduction

Adrenal incidentalomas discovered during abdominal imaging for other indications are not uncommon findings^1^, with a prevalence rising with age from around 3 per cent at the age of 50 years to 10 per cent in the elderly^2^^,^^3^. The diagnostic work-up of adrenal incidentalomas includes endocrine evaluation to evaluate hormone function and radiological imaging to differentiate malignant from benign masses^4^^,^^5^. Identifying malignancy is critical, as the surgical strategy and approach may be influenced by the nature of the mass^5^, such as the choice between laparoscopic and open surgery. Morphological and functional characteristics are often evaluated using combinations of CT, MRI, and [^18^F]fluorodeoxyglucose (FDG) PET–CT. For adrenocortical masses, benign and malignant lesions may potentially be differentiated according to the intracellular lipid content, because adenomas usually have an increased lipid content that gives rise to a low attenuation mean value at unenhanced CT (less than 10 Hounsfield units (HU)), high absolute and relative washout percentages on contrast-enhanced CT (over 60 per cent and more than 40 per cent respectively), and signal drop-off in in-phase and opposition-phase MRI sequences^4^^,^^6^. With large masses, however, heterogeneity in intracellular lipid content, along with areas of necrosis, haemorrhage and calcification, can lead to neither CT nor MRI being able to define the nature of the lesion^1^. As around 10–40 per cent of all adrenal incidentalomas are lipid-poor adenomas, the accuracy of suggested thresholds may be inadequate^7^, potentially leading to surgical overtreatment. Malignant adrenal lesions present with increased metabolic activity^8^ that may be revealed by increased uptake at [^18^F]FDG PET–CT, but the accuracy of the technique remains under debate because of controversies concerning correct cut-off points for maximum standardized uptake values (SUVmax)^9^.

Radiomics is an emerging area of radiology that uses a variety of computational methods to obtain quantitative parameters from CT and MRI, potentially allowing automated analysis even for heterogeneous tumours^10^^,^^11^. Texture analysis is a computational quantitative technique that provides a measure of lesion heterogeneity on the basis of local variations in image brightness, discriminating between pathologically different regions. Statistical texture analysis is based on first- and second-order techniques; the former determine how many pixels have a specific attenuation within a mass, and the latter determine whether those pixels are clustered inside the tumour volume.

Few studies have evaluated radiomic approaches to differentiate between benign and malignant adrenal masses^12^^,^^13^. The aim of this pilot study was to investigate whether CT texture analysis could be a suitable method for distinguishing benign from malignant adrenocortical non-secreting lesions in order to strengthen the indication for surgery, even with an unsupervised machine learning approach.

## Methods

The present study included a selection of patients recruited between 2012 and 2018 at Padua University Hospital, Italy, with indeterminate functionally inactive adrenal lesions discovered incidentally at imaging in patients without other neoplasms.

The study group included patients who underwent adrenalectomy at a single centre and who had an adrenal mass with a mean CT densitometry value in unenhanced acquisition of more than 10 HU and a reduced relative or absolute washout on contrast-enhanced CT (less than 40 per cent and below 60 per cent respectively), without clinically evident and overt laboratory signs of hypercortisolism or virilization, with no radiological signs of local invasion to adjacent structures or distant metastases, and with CT performed by a standardized protocol. A comparator group of non-operated patients diagnosed and followed up at the same centre, with clinically asymptomatic and functionally inactive unilateral adrenal incidentalomas, unequivocal clinical and radiological characteristics of benign behaviour (size less than 4 cm, absence of growth over at least 5 years of follow-up, less than 10 HU on unenhanced CT, absolute and relative washout suggestive of adenoma), and no history of malignancy, was also evaluated. Patients younger than 18 years, those with CT/MRI consistent with myelolipoma, cyst or haematoma, and patients with incomplete or unavailable CT imaging were excluded.

The study was performed in accordance with the guidelines in the Declaration of Helsinki and was approved by the Padua University Hospital Ethics Committee (Protocol 0042599); all patients gave informed consent.

All patients underwent clinical and hormonal evaluation, according to the European Society of Endocrinology Clinical Practice Guidelines^1^. Histopathological examinations in operated patients were performed by a dedicated pathologist^14^.

### Images acquisition and analysis

All CT examinations were performed with a 128-slice scanner (Somatom Definition™; Siemens Healthineers, Erlangen, Germany), including unenhanced and contrast-enhanced scans after intravenous injection of 2 ml/kg iohexol 350 mg iodine per ml (Omnipaque™; GE Healthcare, Milwaukee, Wisconsin, USA) followed by a 50-ml saline flush. There was craniocaudal image acquisition with 120 kV tube voltage, 250 mAs effective dose, 0.5 s rotation time, 0.6 mm detector collimation, and 0.75 pitch. Slice thickness was 5 mm for unenhanced acquisition and 3 mm for arterial and venous scans; all images analysed had soft-tissue reconstruction with a 30B kernel. The arterial-phase image was acquired 15 s after the achievement of 100 HU within the aortic lumen, and the venous-phase image 80 s after contrast administration. The mean densitometry value in unenhanced, venous, and delayed phases was calculated by drawing a region of interest (ROI) along the margins of the maximum diameter of the adrenal mass in the axial slice.

Absolute and relative washouts were calculated as reported previously^15^. When [^18^F]FDG PET–CT was available, SUVmax was assessed as described previously^8^.

**Fig. 1:**
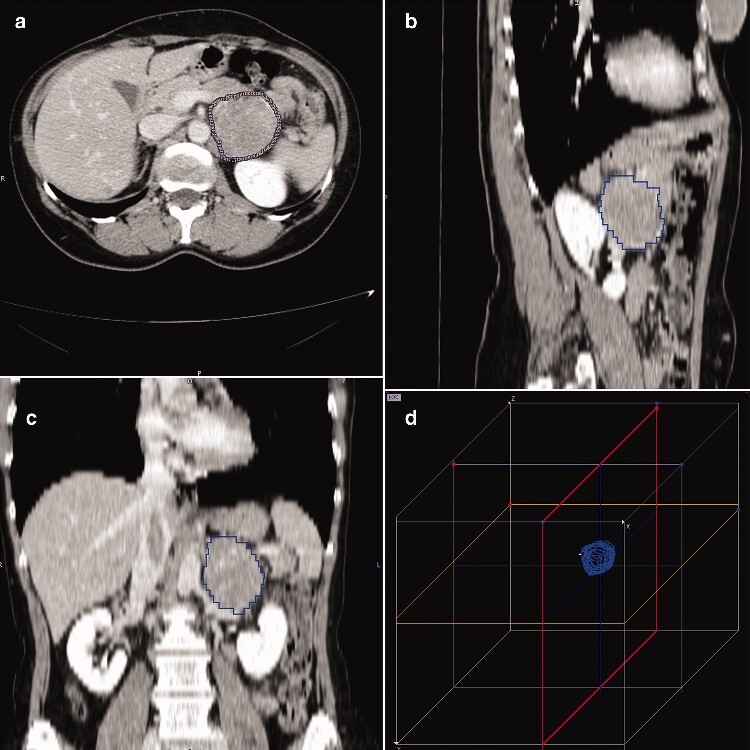
Volume of interest of an adrenal mass delineated using PMOD software Volume of interest of adrenal mass on venous-phase CT: **a** axial, **b** sagittal, and **c** coronal views, and **d** volume rendering reconstruction.

In each CT slice of the three phases (unenhanced, arterial, and venous), two radiologists in consensus drew a ROI along the margin of the adrenal lesion using PMOD (PMOD Group, Zurich, Switzerland), a specific software for image texture analysis. A volume of interest (VOI) for each lesion in each phase was created (*Fig. 1*). Each voxel inside the VOIs measured 0.744 × 0.744 × 5.000 mm for unenhanced scans, and 0.742 × 0.742 × 3.000 mm for contrast-enhanced acquisitions. Densitometry data in HU for each voxel inside the VOIs were extracted automatically by the software, which obtained 32 radiomic features from the image texture of each VOI. The radiomic features analysed were five first-order parameters, and 27 second-order grey-level co-occurrence matrix (GLCM) and second-order run-length matrix (RLM) parameters^16^, as described in detail in *Table S1* and *Fig. S1*.

**Fig.2:**
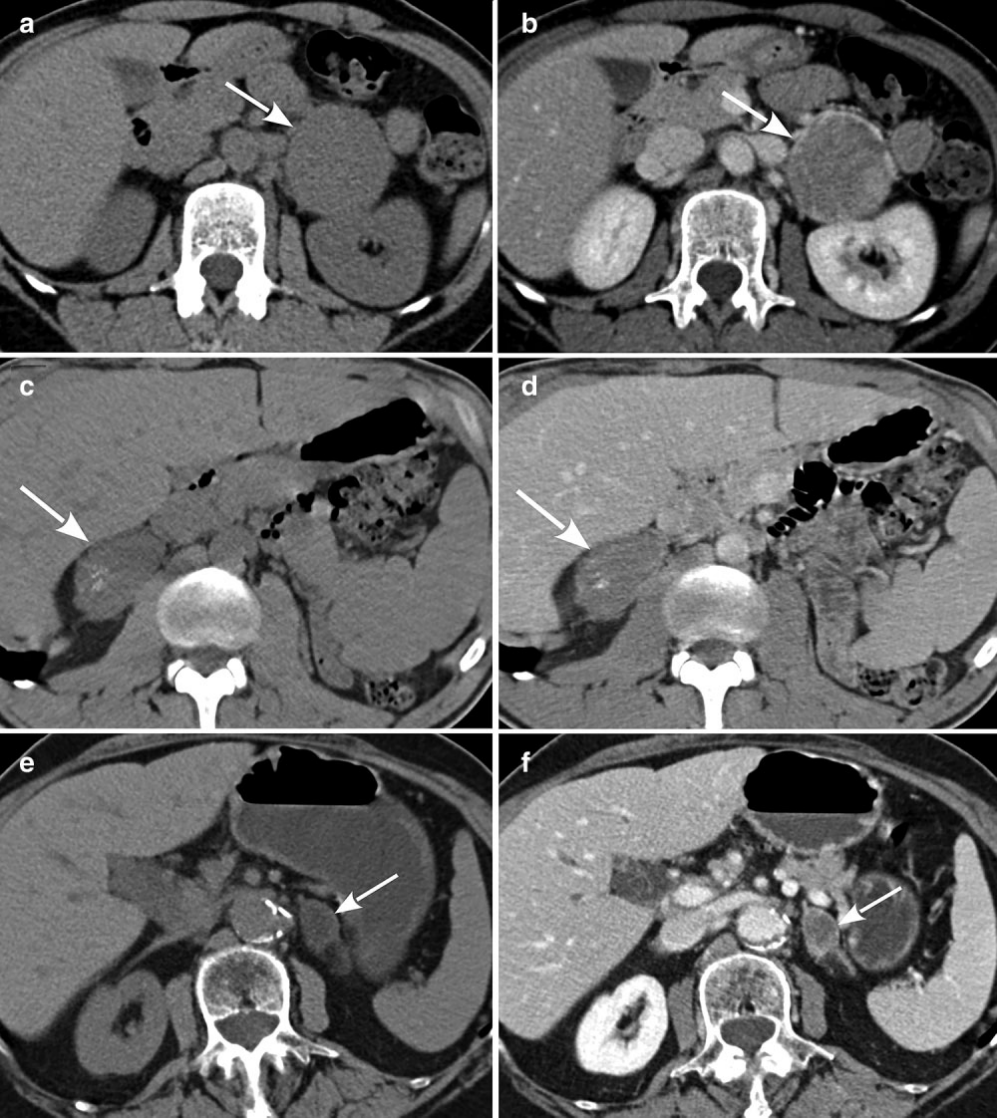
Axial CT images of an adrenocortical carcinoma, adenoma, and adrenal incidentaloma in unenhanced and venous phase Unenhanced (**a**,**c**,**e**) and venous-phase images (**b**,**d**,**f**) of **a**,**b** adrenocortical carcinoma (34 Hounsfield units (HU); relative washout less than 40 per cent), **c**,**d** adenoma (33 HU; relative washout less than 40 per cent), and **e**,**f** adrenal incidentaloma (–5 HU; relative washout over 40 per cent).

**Fig. 3:**
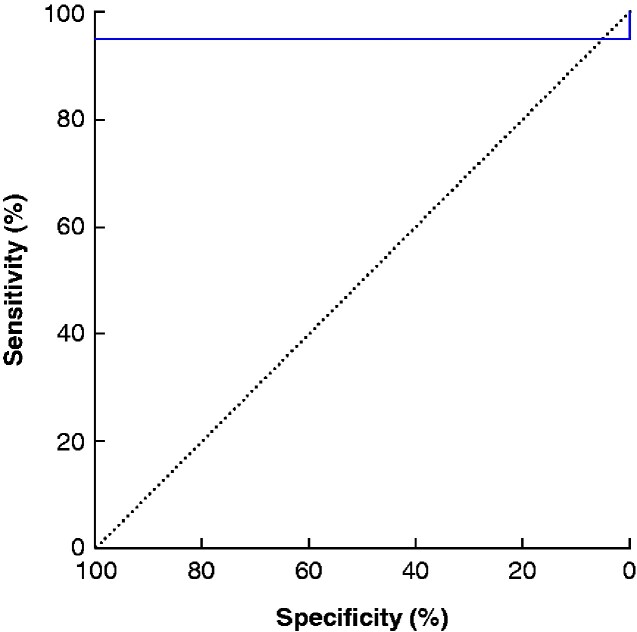
Receiver operating characteristic (ROC) curve testing for histogram mean in unenhanced CT image to distinguish adrenocortical carcinoma *versus* adenoma and adrenal incidentaloma At Youden index analysis, the cut-off of 22.5 Hounsfield units showed a sensitivity of 95 per cent and a specificity of 100 per cent.

### Statistical analysis

Data from adenomas, carcinomas, and patients with unoperated incidentalomas were compared using χ^2^ and Student’s *t* tests, as appropriate. The level of significance was taken as *P* < 0.050.

Principal component analysis (PCA)^17^ was applied to the radiomic features extracted by dedicated software. PCA is a mathematical transformation providing a set of new components (principal components), which are obtained by a linear combination of the original ones. Principal components are ordered according to their contribution to the total variation of the data set. Features that had *P* < 0.050 in the differentiation between adrenocortical carcinoma and the other two groups were selected. A receiver operating characteristic (ROC) curve was built for each parameter selected, and the area under the ROC curve, and sensitivity and specificity at Youden’s index, were calculated. Data for the lesions were analysed using a K-means clustering technique^18^, an unsupervised machine learning approach. The detection rate of adrenocortical carcinoma *versus* adenoma and incidentaloma was calculated, regardless of pretest classification of the disease. Statistical analysis was done using R software (R Foundation for Statistical Computing, Vienna, Austria).

## Results

From a database of 336 operated patients, 19 (9 adenomas, 10 carcinomas) met the inclusion criteria and were included in the study. In accordance with the selection criteria, all patients with adrenocortical carcinomas had disease confined to the gland (stage I–II according to European Network for the Study of Adrenal Tumours classification^19^). Eleven patients comprised the unoperated cohort of adrenal incidentalomas. Clinical characteristics of the patients and imaging features of the tumours are summarized in *Table 1*. The mean size of the adrenal lesions was significantly larger for carcinomas and adenomas compared with incidentalomas (mean(s.d.) 62.3(35.2) *versus* 25.9(1.4) mm, *P* = 0.003; 56.6(42.4) *versus* 25.9(1.4) mm, *P* = 0.027).

**Table 1:** Clinical and radiological features of patients evaluated for adrenal mass

	**ACC** **(*n* = 10)**	**Adenoma** **(*n* = 9)**	**AI** **(*n* = 11)**	*P* ^§^		
				ACC *versus* adenoma	ACC *versus* AI	Adenoma *versus* AI
Age (years)*	53.5(14.4)	66.2(6.4)	71.4(9.4)	0.537	0.042	0.246
**Sex ratio (M : F)**	4 : 6	4 : 5	5 : 6	0.265^¶^	0.490^¶^	0.516^¶^
**Lesion side**				0.625^¶^	0.715^¶^	0.854^¶^
Right	6	5	4			
Left	4	4	7			
Axial maximum diameter (mm)*	62.3(35.2)	56.6(42.4)	25.9(1.4)	0.753	0.003	0.027
Mean densitometry value (HU)*	33.4(4.7)	20.2(9.2)	2.8(9.4)	0.001	< 0.001	0.001
Relative washout (%)*	16.5(0.2)	23.7(0.4)	46.5(0.5)	< 0.001	< 0.001	< 0.001
Absolute washout (%)*	30.0(0.2)	32.2(0.1)	63.3(0.3)	< 0.001	< 0.001	< 0.001
SUVmax*	14.9(7.2)^†^	15.8(22.2)^‡^	–	0.905	–	–

* Values are mean(s.d.). PET–CT performed in

† six and

‡ four patients. ACC, adrenocortical carcinoma; AI, adrenal incidentaloma; HU, Hounsfield units; SUVmax, maximum standardized uptake value.

§ Student’s *t* test, except

¶ χ^2^ test.

In accordance with the selection criteria, mean unenhanced densitometry, and relative and absolute washout values for both carcinomas and adenomas were suggestive of malignancy (more than 10 HU, and less than 40 per cent and below 60 per cent respectively), although higher unenhanced values and lower washout values were detected in carcinomas. Values for the incidentaloma group were always indicative of benign lesions (*Fig. 2*). [^18^F]FDG PET–CT was available for 10 patients (6 carcinoma, 4 adenoma), and mean SUVmax was no different between carcinomas and adenomas (mean(s.d.) 14.9(7.2) *versus* 15.8(22.2); *P* = 0.905).

Texture analysis showed that two first-order features in unenhanced CT and one in arterial CT, and 14 second-order parameters in unenhanced and venous CT and 10 in arterial CT, were able to differentiate adrenocortical carcinomas form adenomas and incidentalomas (*P* < 0.050).

Among the first-order parameters, the histogram mean in unenhanced CT acquisition showed a sensitivity of 95 per cent and specificity of 100 per cent using the identified Youden index cut-off of 22.5 HU (*Fig. 3*). Specificity and sensitivity of 90 and 50 per cent respectively for the histogram mean in arterial-phase CT acquisition was also observed. Histogram entropy in the unenhanced CT phase had a specificity of 90 per cent and a sensitivity of 65 per cent.

In the analysis of second-order features, a specificity of 100 per cent and a sensitivity of 95 per cent was found for 10 parameters in unenhanced CT images (GLCM autocorrelation, entropy, difference entropy, sum average, sum entropy, maximum probability, homogeneity inverse difference moment, RLM long run low grey-level emphasis, short run high grey-level emphasis, grey-level non-uniformity), in eight features of arterial-phase images (GLCM autocorrelation, entropy, difference entropy, sum average, sum entropy, maximum probability, homogeneity inverse difference moment, RLM short run high grey-level emphasis), and in 10 parameters for venous-phase CT images (GLCM autocorrelation, entropy, difference entropy, sum average, sum entropy, maximum probability, homogeneity inverse difference moment, RLM long run low grey-level emphasis, short run high grey-level emphasis, grey-level non-uniformity). A specificity of 100 per cent and sensitivity of 90 per cent for RLM short run emphasis in unenhanced images, and RLM long run low grey-level emphasis in arterial acquisitions was observed. A specificity of 100 per cent and sensitivity of 85 per cent was found in three second-order features in the venous phase. The specificity was 90 per cent and sensitivity 85 per cent for RLM short run low grey-level emphasis in unenhanced images and RLM run percentage in venous acquisitions, specificity 90 per cent and sensitivity 95 per cent for RLM low grey-level emphasis in unenhanced acquisitions, specificity 90 per cent and sensitivity 50 per cent for RLM run percentage in unenhanced images, and specificity 90 per cent and sensitivity 20 per cent for RLM run percentage in the arterial phase.

**Fig. 4:**
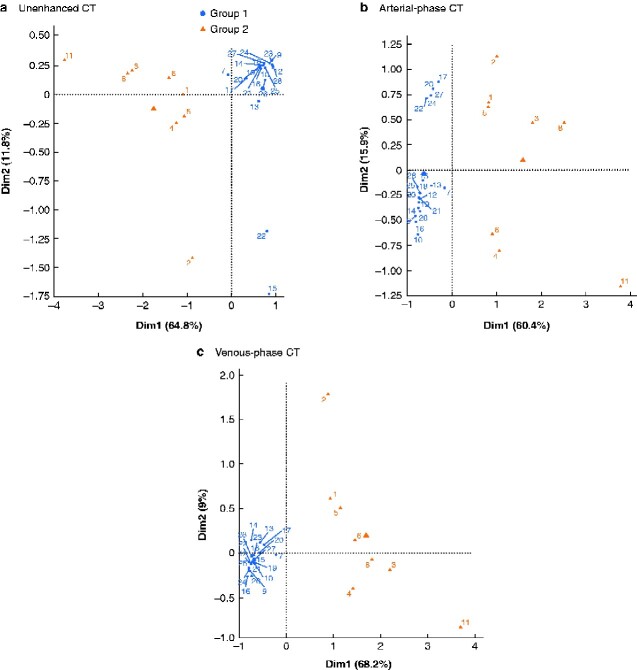
K-means algorithm clustering of unenhanced, arterial-phase, and venous-phase CT acquisitions **a** Unenhanced, **b** arterial-phase, and **c** venous-phase CT acquisitions. Representation of data with respect to the first (*x*-axis; dim1) and second (*y*-axis; dim2) principal components is provided by principal component analysis, explaining 64.8 and 11.8 per cent (unenhanced CT), 60.4 and 15.9 per cent (arterial-phase CT), and 68.2 and 9.0 per cent (venous-phase CT) of the data variance respectively. Each dot represents a subject and is colour-coded on the basis of the clustering analysis. Larger markers represent the centre of mass of the two clusters. Group 1, subjects classified as having a benign adrenal mass (adenoma and adrenal incidentaloma); group 2, subjects classified as having adrenocortical carcinoma.

Using the whole data set for each phase (unenhanced, arterial, and venous), a K-means analysis was undertaken to search for any clustering of subjects. Two adrenocortical carcinomas were outliers compared with the distribution of the other patients (potentially owing to massive calcifications compared with other carcinomas) and these were removed from the analysis. The results of the K-means algorithm for the three phases are reported in *Fig. 4*. Two clusters of patients were identified for each acquisition; the detection rate was seven of eight for adrenocortical carcinoma, and 19 of 20 for adrenal adenoma and incidentaloma in all three phases.

## Discussion

Adrenal incidentalomas are frequent unexpected findings in imaging studies^1^^,^^4^. After excluding hormone hypersecretion, the critical point for each incidentally detected adrenal mass is to establish whether it is benign or malignant, in order to assess the further strategy and potential surgical approach.

Several features have been studied to predict the nature of adrenal mass at imaging^4^^,^^6^, but inaccuracy is often reported. Although size alone is often used as an indicator of the risk of malignancy (4 cm is generally adopted as first cut-off), overlap between benign and malignant lesions can result in unnecessary surgery for diagnostic purposes^1^.

Increased attenuation values equal to or higher than 10 HU on unenhanced CT, indicating a lipid-poor adrenal lesion, have a high sensitivity (100 (95 per cent c.i. 91 to 100) per cent) but poor specificity (72 (60 to 82) per cent)^9^^,^^20^ for detecting malignancy. A cut-off of 20 HU has been proposed as a threshold for differentiating between benign and malignant adrenal masses^7^^,^^21^^,^^22^. The accuracy of this threshold is supported by the present study , where a sensitivity of 95 per cent and specificity of 100 per cent were found when the cut-off mean densitometry value of 22.5 HU was used for CT texture analysis. Absolute and relative washout in delayed-phase CT images has also been reported to predict malignancy^23^, but sensitivity and specificity in incidentally detected adrenal masses have been evaluated in only a few studies using different protocols^1^^,^^9^. MRI with chemical shift imaging has provided limited evidence^9^ and SUVmax at [^18^F]FDG PET–CT has limited accuracy (overall sensitivity and specificity 86.7 and 86.1 per cent respectively)^8^. In addition, image analysis of large adrenal masses poses a challenge owing to the presence of necrotic and haemorrhagic areas as well as calcifications.

Image-based texture analysis (radiomics) allows quantitative parameters to be obtained from conventional images, even in the analysis of large heterogeneous tumours^10^^,^^11^. Texture analysis has been applied to many organs, including brain, lung, liver, stomach, pancreas, kidney, and soft tissue to differentiate benign from malignant lesions^24^. Only two studies^12^^,^^13^ have focused on primary adrenocortical lesions.

One small series^12^ found that the mean values for 18 textural features on contrast-enhanced CT and nine on unenhanced CT differed significantly between lipid-poor adenomas and adrenal malignancy, whereas the other^13^, which included 29 adrenocortical carcinomas and 25 adenomas larger than 4 cm, reported an accuracy of CT texture analysis in predicting malignancies of 61–76 per cent. The latter series, however, also included hypersecreting adrenal masses and the predictive model using CT texture analysis combined with CT attenuation values achieved a diagnostic accuracy of only 82 per cent.

The present study focused on indeterminate non-secreting and clinically asymptomatic incidentally discovered adrenocortical tumours, where conventional radiology and PET–CT SUVmax were unable to clearly differentiate adenomas from carcinomas. In this study, radiomic texture analysis was able to differentiate between benign and malignant adrenal masses. Several parameters among first- and second-order features had high diagnostic accuracy (sensitivity and specificity over 90 per cent), and may therefore be suitable for clinical purposes. Several unenhanced CT textural parameters also may be used to distinguish adenoma from carcinoma. The advantage of extracting images from the unenhanced CT image is that this is easier to control, imaging is easier to obtain, and the texture data extracted depend only on the inherent density of the tumour. On the other hand, enhanced CT images are affected by many factors, including differences in contrast materials and injection rates, as well as the scan delay time. For these reasons, only data from the same CT scanner acquired at the same institution with the same protocol were included in the present study.

Radiomics allows extracted quantitative data to be used as the input to a machine learning process. Combining data and using a machine learning unsupervised approach, two clusters of patients were clearly identified, achieving a detection rate of seven of eight for adrenocortical carcinoma and nine of ten for adenoma. Only one patient with malignancy would have been misclassified as having an adenoma with this machine learning-based approach. Conversely, one large adenoma would have been categorized as malignant. With a classical radiological approach, all of these lesions would have been classified as potentially malignant.

The present study has limitations related to its design and the relatively small sample size, reflecting the strict inclusion criteria (indeterminate, incidentally discovered non-functional asymptomatic adrenal masses, studied at the same institution with the same imaging protocol). Despite such limitations, these preliminary data indicate that radiomics might be a useful tool in differentiating benign from malignant adrenocortical masses, thus limiting the number of diagnostic adrenalectomies.

## Supplementary Material

zraa061_Supplementary_Data

## Notes

Accepted for presentation to the Nineth Biennial Congress of the European Society of Endocrine Surgeons, Athens, Greece, May 2020
