# Supplementary material for: Radiomics: a new tool to differentiate adrenocortical adenoma from carcinoma
Source: BJS Open. 2021 Mar 3;5(1):zraa061. doi: 10.1093/bjsopen/zraa061 (PMC7937424; doi:10.1093/bjsopen/zraa061)
Supplement: zraa061_Supplementary_Data [file zraa061_supplementary_data.zip › Supplementary_material_2.docx]

**Supplementary data**

**Table S1.** First and second-order texture parameters analyzed.

|  | **PARAMETER** | **DEFINITION** |
| --- | --- | --- |
|  | Mean | average of a range of values |
|  | Variance | [expectation](https://en.wikipedia.org/wiki/Expected_value) of the squared [deviation](https://en.wikipedia.org/wiki/Deviation_(statistics)) of a [random variable](https://en.wikipedia.org/wiki/Random_variable) from its [mean](https://en.wikipedia.org/wiki/Expected_value) |
|  | Skewness | measure of the asymmetry of the [probability distribution](https://en.wikipedia.org/wiki/Probability_distribution) of a [real](https://en.wikipedia.org/wiki/Real_number)-valued [random variable](https://en.wikipedia.org/wiki/Random_variable) |
| **First-order** | Kurtosis | measure of the "tailedness" of the [probability distribution](https://en.wikipedia.org/wiki/Probability_distribution) of a [real](https://en.wikipedia.org/wiki/Real_number)-valued [random variable](https://en.wikipedia.org/wiki/Random_variable) |
|  | Entropy | Measure o fan image’s smoothness in terms of gray-level values |
|  | **Gray-level co-occurrence matrix (GLCM)** | Mathematical procedure that provides a number of second-order statistics relating to the grey level relationships in a neighborhood around a pixel |
|  | GLCM Energy | Returns the sum of squared elements in the GLCM |
|  | GLCM Contrast | Measure of the intensity contrast between a pixel and its neighbor over the whole image |
|  | GLCM Correlation | Measure of how correlated a pixel is to its neighbor over the whole image |
| **Second-order** | GLCM Variance | Sum of the squares of the differences between the intensity of the central pixel and its neighbors |
|  | GLCM inverse different moment | Measure of local homogeneity of an image |
|  | GLCM sum average | Measures the mean of the grey level sum distribution of the image |
|  | GLCM sum variance | Measures the dispersion (with regard to the mean) of the grey level sum distribution of the image |
|  | GLCM sum entropy | Measures the disorder related to the grey level sum distribution of the image |
|  | GLCM difference average (or dissimilarity) | Measures the mean of the gray level difference distribution of the image |
|  | GLCM difference variance | Measure of heterogeneity that places higher weights on differing intensity level pairs that deviate more from the mean |
|  | GLCM difference entropy | Measures the disorder related to the grey level difference distribution of the image |
|  | GLCM auto-correlation | Measure of the magnitude of the fineness and coarseness of texture |
|  | GLCM cluster shade | Measures skewness of the GLCM matrix and is believed to gauge the perceptual concepts of uniformity |
|  | GLCM cluster prominence | Measure asymmetry of the GLCM |
|  | GLCM maximum probability | Measure of the occurrences of the most predominant pair of neighboring intensity values |
|  | GLCM inverse difference | Measure of local homogeneity of an image |
|  | **Run length matrix (RLM)** | The number of runs with pixels of a fixed grey level and run length |
|  | RLM short run emphasis | Measures the distribution of short runs |
|  | RLM long run emphasis | Measures the distribution of long runs |
|  | RLM low grey level run emphasis | Measure of the distribution of the low grey level runs |
|  | RLM high grey level run emphasis | Measure of the distribution of the high grey level runs |
|  | RLM grey level non uniformity | Measure of the non-uniformity of the grey levels |
|  | RLM run length non uniformity | Measure of the non-uniformity of the length of the homogeneous runs |
|  | RLM run percentage | Measures the homogeneity of the homogeneous runs |
|  | RLM short run low grey level emphasis | Measure of the distribution of the short homogeneous runs with low grey levels |
|  | RLM long run high grey level emphasis | Measure of the distribution of the long homogeneous runs with high grey levels |
|  | RLM short run high grey level emphasis | Measure of the distribution of the short homogeneous runs with high grey levels |
|  | RLM long run low grey level emphasis | Measure of the distribution of the long homogeneous runs with low grey levels |

First-order statistics describes the distribution of pixels in the VOI using image histogram (pixel occurrence probability) to calculate texture with standard descriptors (mean, variance, skewness, kurtosis, and entropy).

Second-order texture parameters describe how many neighbor pixels have the same grey-level and their relationship in the image. **The Grey level co-occurrence matrix (GLCM)** represents the joint probability of certain sets of pixels having certain grey-level values. It calculates how many times a pixel with grey-level *i* occurs jointly with another pixel having a grey value *j*. By varying the displacement vector *d* between each pair of pixels, many GLCMs with different directions can be generated. From GLCM 16 features were generated, which are: Energy, Contrast, Correlation, Variance, inverse different moment, sum average, sum variance, sum entropy, difference average (or dissimilarity), difference variance, difference entropy, informational measure of correlation (1 and 2), auto-correlation, cluster shade, cluster prominence, maximum probability, inverse difference.

The **grey level run-length matrix (RLM)** is defined as the numbers of runs with pixels of gray level i and run length j for a given direction θ. The following 11 features were derived: short run emphasis, long run emphasis, low grey level run emphasis, high grey level run emphasis, grey level non uniformity, run length non uniformity, run percentage, short run low grey level emphasis, long run high grey level emphasis, short run high grey level emphasis, long run low grey level emphasis.

**Supplementary Figure Legend**

**Figure 1S.** Schematic representation of texture analysis and the machine learning approach performed in this study.

CT=computed tomography; ROI=region of interest; VOI=volume of interest; GLCM=grey level color matrix; RLM=run length matrix.
