# Supplementary figures and images for: Radiomics: a new tool to differentiate adrenocortical adenoma from carcinoma
Source: BJS Open. 2021 Mar 3;5(1):zraa061. doi: 10.1093/bjsopen/zraa061 (PMC7937424; doi:10.1093/bjsopen/zraa061)

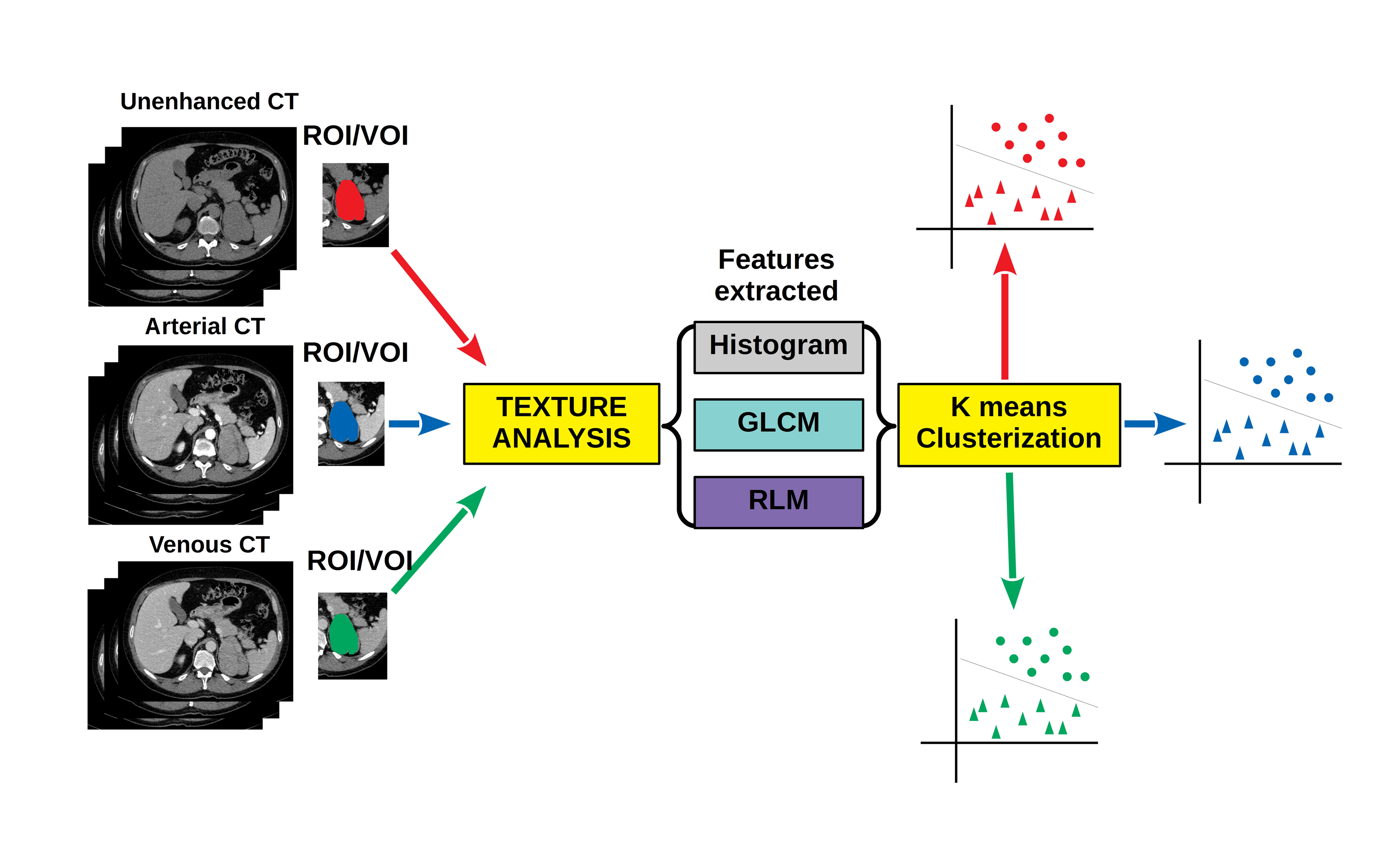

Supplement: zraa061_Supplementary_Data [file zraa061_supplementary_data.zip › Figure S1_IacoboneMaurizio.jpg]
